# Assessing the Functional and Structural Stability of the Met80Ala Mutant of Cytochrome *c* in Dimethylsulfoxide

**DOI:** 10.3390/molecules27175630

**Published:** 2022-08-31

**Authors:** Giulia Di Rocco, Antonio Ranieri, Marco Borsari, Marco Sola, Carlo Augusto Bortolotti, Gianantonio Battistuzzi

**Affiliations:** 1 Department of Life Sciences, University of Modena and Reggio Emilia, Via Campi 103, 41125 Modena, Italy; 2 Department of Chemical and Geological Sciences, University of Modena and Reggio Emilia, Via Campi 103, 41125 Modena, Italy

**Keywords:** cytochrome *c*, dimethylsulfoxide, electron transfer, protein unfolding

## Abstract

The Met80Ala variant of yeast cytochrome *c* is known to possess electrocatalytic properties that are absent in the wild type form and that make it a promising candidate for biocatalysis and biosensing. The versatility of an enzyme is enhanced by the stability in mixed aqueous/organic solvents that would allow poorly water-soluble substrates to be targeted. In this work, we have evaluated the effect of dimethylsulfoxide (DMSO) on the functionality of the Met80Ala cytochrome *c* mutant, by investigating the thermodynamics and kinetics of electron transfer in mixed water/DMSO solutions up to 50% DMSO *v*/*v*. In parallel, we have monitored spectroscopically the retention of the main structural features in the same medium, focusing on both the overall protein structure and the heme center. We found that the organic solvent exerts only minor effects on the redox and structural properties of the mutant mostly as a result of the modification of the dielectric constant of the solvent. This would warrant proper functionality of this variant also under these potentially hostile experimental conditions, that differ from the physiological milieu of cytochrome *c*.

## 1. Introduction

**Figure 1:**
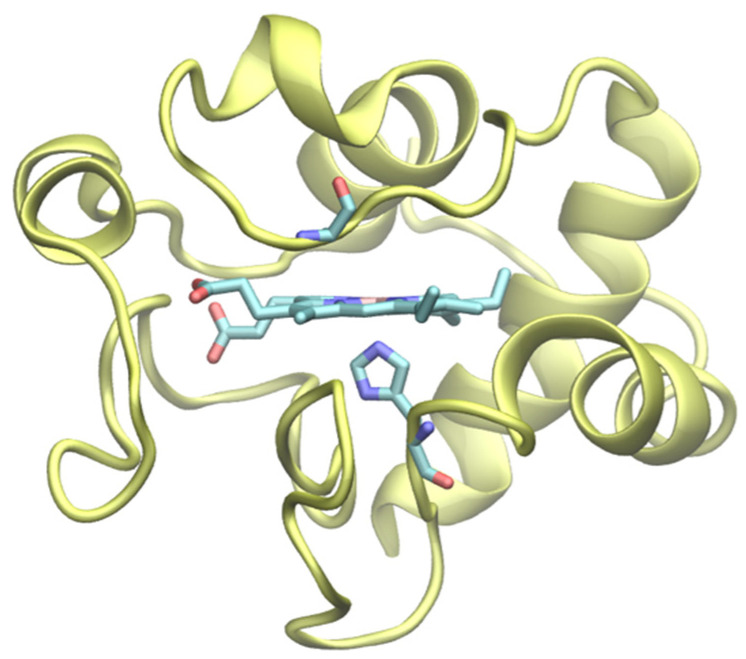
Cartoon representation of the 3D structure of the Met80Ala mutant of yeast cytochrome *c* (PDB: 1FHB). The heme, the iron axial ligand His18 and the non-coordinating Ala80 are represented as licorice.

Cytochrome *c* (cytc hereafter) is among the most widely investigated electron transfer (ET) proteins [1,2,3,4,5,6,7,8,9]. Being one of the main characters in a highly relevant physiological process such as mitochondrial respiration, cytc has served as a paradigmatic system for elucidating the fundamentals of biological redox processes [10,11,12,13] and has been the target of several experimental [10,14,15,16,17] and computational [18,19,20,21] investigations. Besides its role as ET species, though, cytc also plays a key role in cell apoptosis [22]. The discovery of the moonlighting nature of cytc [1,4,22,23,24,25,26,27] has gathered further attention from the scientific community on this small globular protein, with the aim of endowing it with non-native catalytic properties either by changes in environmental conditions or by protein engineering [28,29,30]. These properties would benefit from a few notable features of cytc such as the stability in a wide range of pH and temperature values, the high yield of recombinant expression, and the ability to retain its functionality upon adsorption onto functionalized surfaces. In particular, the Met80Ala variant of yeast cytc [31,32,33,34] has been found previously to act as a versatile engineered enzyme able to catalytically reduce a number of substrates (nitrite, dioxygen, hydrogen peroxide) while maintaining some of the most desirable features of the native species [28,29,35,36,37,38,39]. In this mutant, a non-coordinating alanine residue replaces the methionine at position 80 acting as an axial iron ligand in the wt protein, without inducing significant structural rearrangements (Figure 1). Despite the mutation, the Fe^3+^ is still hexacoordinated, since the free axial coordination position is occupied by a hydroxide anion that protonates with a pK_a_ of 5.6 [31,32,34,35,40,41,42,43]. The latter could easily be replaced by small neutral and anion ligands, such as dioxygen, hydrogen peroxide, and nitrite [28,29,35,36,37,38,39,44]. One of the limitations to the widespread use of enzymes to replace solid-state catalysts is their limited stability in non-aqueous solvents. The possibility of performing catalysis in mixed aqueous/organic solvent would open the possibility of considering substrates that are poorly soluble in water, thus expanding the portfolio of molecules that can be enzymatically transformed by (metallo)proteins. For this reason, exploring the structural and functional stability of native or engineered enzymes upon changes in the medium composition is crucial to assess their potential use for several applications ranging from biosensing to (electro)biocatalysis and molecular switches [17,30,44,45,46,47,48,49,50,51,52,53,54,55,56].

In this work, we characterized the redox properties of the Met80Ala variant of cytc in water/dimethylsufloxide (DMSO) mixtures up to 50% *v*/*v* DMSO, investigating how the thermodynamics and kinetics of the electron transfer for the freely diffusing protein are affected by the increasing presence of the organic solvent, which sensibly influences the dielectric properties of the medium. Indeed, the available data indicate that the dielectric constant of H_2_O/DMSO solutions containing DMSO mole fractions of 0.1 and 0.2 (roughly corresponding to 30% and 50% *v*/*v* DMSO) are between 76 and 77 and 71 and 74, respectively [57,58]. We then explored how the changes in the protein functionality of redox species are linked to structural modifications, by performing extensive spectroscopic characterization under the same experimental conditions. The results presented in this work were then compared to those recently obtained by us in the same mixed water/DMSO solvent for the electrode-immobilized M80A variant [44], allowing information to be gained on how surface confinement of the protein impacts the response of the biomolecule to varying medium composition.

## 2. Results and Discussion

### 2.1. Effect of DMSO on the Reduction Thermodynamics of the M80A Cytc Mutant

**Figure 2:**
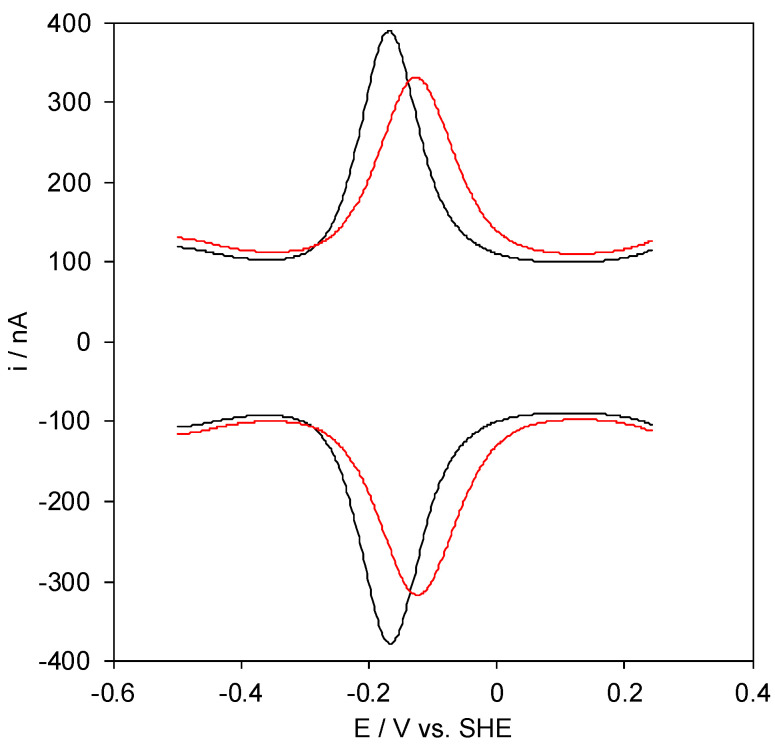
Cathodic and anodic Square Wave Voltammograms of the M80A cytc variant in 0% (black line) and 50% (red line) DMSO. Base electrolyte: 5 mM sodium phosphate buffer pH 7 plus 100 mM sodium perchlorate, T = 293 K.

The electrochemical response of the M80A cytc mutant (M80A hereafter) to the presence of organic solvent was investigated by recording square wave voltammograms in DMSO/water binary mixtures containing DMSO volumes up to 50%. Independently of the DMSO/water ratio, only a single signal can be observed both in the cathodic and anodic scans (Figure 2), which can be attributed to reversible protein-electrode electron transfer.

**Figure 3:**
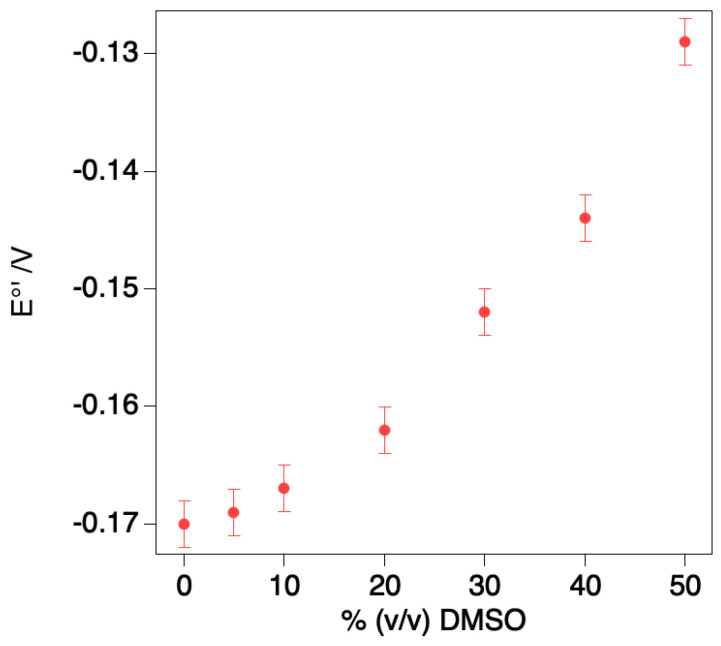
Dependence of the standard reduction potential E°′ on the % DMSO for the freely diffusing M80A variant of yeast cytc.

As shown in Figure 3, an increase in DMSO concentration up to 50% *v*/*v* shifts the E°′ value of M80A towards less negative values by about 40 mV. The E°′ vs. DMSO concentration plot is approximately biphasic, with a larger slope observed for DMSO concentration > 20%.

DMSO was reported previously to induce a similar anodic E°′ shift (although less pronounced, +23 mV at 50% DMSO) also for the M80A variant immobilized on an anionic mercaptoundecanoic acid (MUA)/mercaptoundecanol (MU) self-assembled monolayer (SAM) [44]. Interestingly, the DMSO-induced E°′ change found here is opposite to that observed for wt cytc in solution, whose E°′ shifted cathodically (−15 mV at 50% DMSO) [56].

The fact that the DMSO-induced change in E°′ found here is modest suggests that only minor solvent-induced conformational changes occur, probably due to the change in the dielectric constant of the medium. The anodic shift indicates that the presence of the organic solvent selectively stabilizes the reduced over the oxidized form, in line with what is expected by a dielectric constant decrease which should stabilize the less positively charged ferrous form.

The reaction entropy (ΔS°′_rc_) and enthalpy (ΔH°′_rc_) for the reduction of ferriheme, were obtained from the E°′ vs. T profiles measured at 0, 10, 20, 30, 40, and 50% DMSO (Figure S1 and Table 1).

The reduction thermodynamics shows a marked dependence on the water/DMSO ratio. In particular, ΔS°′_rc_ is positive and increases with increasing DMSO concentration, thus favoring the reduced form. ΔS°′_rc_ is determined by reduction-induced changes in the number of accessible configurational microstates that are known to be mostly due to changes in the H-bonding network of the water molecules within the protein solvation sphere, the so-called solvent reorganization effects [59], although minor contributions from changes in the protein secondary/tertiary structure or chain flexibility cannot be excluded. Here, the positive ΔS°′_rc_ values are consistent both with the significant solvent accessibility of the metal center, since the decreased electrostatic interaction of the ferrous heme with the solvent molecules in the heme cavity should lead to a reduction-induced decrease in ordering, as well as with the detachment of the sixth axial OH^−^ ligand upon Fe^3+^ reduction [28,35,36]. As the latter is involved in H-bonding interactions with the solvent molecules in the heme cavity, its release from the Fe^2+^ coordination sphere should weaken the H-bonding network contributing to the reduction-induced decrease in the ordering of the solvent molecules. In this vein, the ΔS°′_rc_ increase with increasing DMSO concentration could be the result of an enhanced disorder in the H-bond network in the heme pocket following Fe^3+^-reduction caused by an increased content of organic solvent.

**Figure 4:**
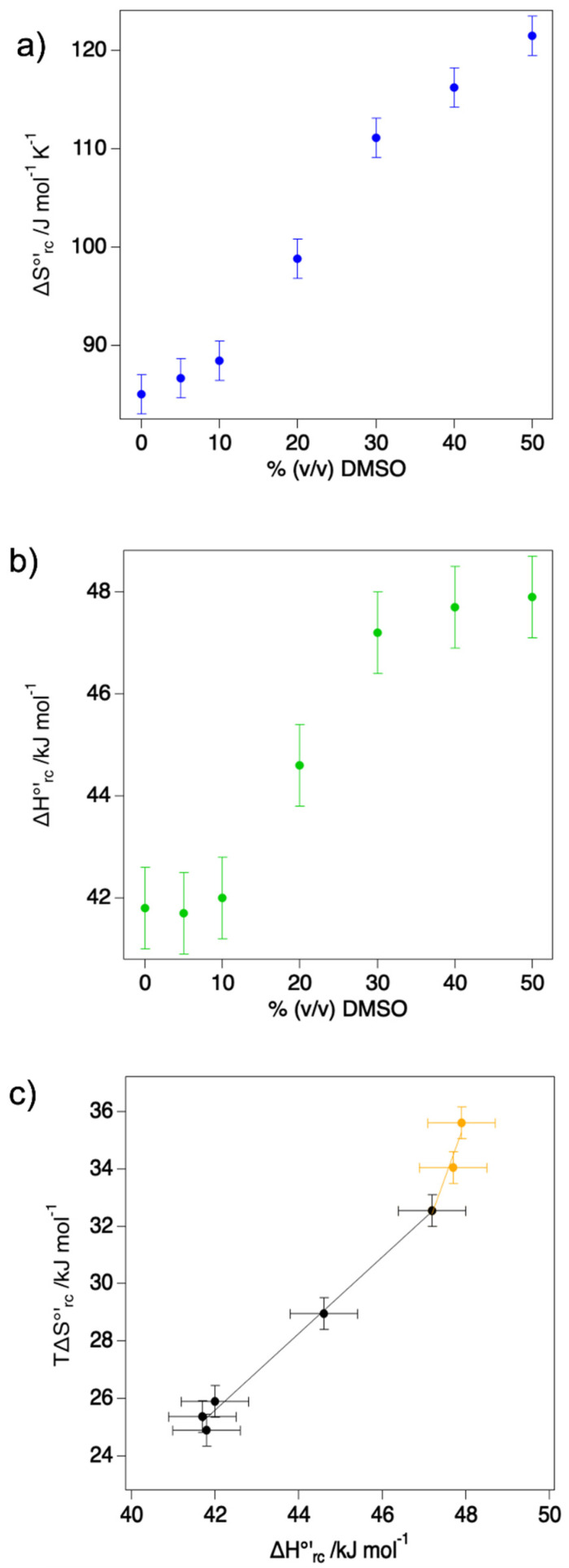
Dependence of ΔS°′_rc_ (**a**) and ΔH°′_rc_ (**b**) on the %DMSO for M80A mutant of yeast cytc. (**c**) Enthalpy-entropy compensation plot. The temperature considered to calculate the entropic component was 293 K. The black and orange lines are least-squares fits to the data points for %DMSO ≤ 30% and %DMSO ≥ 30%, respectively.

ΔH°′_rc_ is instead determined by first and second coordination sphere effects, the former being related to the nature of the donor atoms and the coordination geometry, and the latter including electrostatic interactions between the charge of the metal center and either dipole (provided by the protein and/or the solvent) or net surface charges [59]. Here, ΔH°′_rc_ is also positive and increases with DMSO concentration, indicating an enthalpic stabilization of the oxidized form. As the DMSO-induced changes in ΔS°′_rc_ and ΔH°′_rc_ exert opposing effects on E°′, the observed anodic shift in the reduction potential upon increasing DMSO concentration is due to the prevalence of the former contribution. As shown in Figure 4, both ΔH°′_rc_ and ΔS°′_rc_ follow a sigmoidal trend, which is in contrast with the behavior observed previously for the same variant adsorbed on MUA/MU, which showed a linear dependence on DMSO concentration in the 0–50% range [44].

Further insight into the molecular determinants of the reduction thermodynamics in water/organic mixture can be gained from a compensation plot (Figure 4c), in which the entropic contribution to the reduction of free energy is plotted vs. the enthalpic term [59]. Enthalpy/entropy compensation (EEC, also indicated as H-S) [60,61] has often been observed in the reduction processes involving electron transfer metalloproteins [49,59,62,63,64,65], and one of the main consequences of this counterbalancing effect is to limit (or to offset in some cases) the role of the entropic term in defining the overall E°′ value. Here, a clear H-S compensation can be observed up to 30% DMSO, while a strong change in the slope of the linear correlation occurs for larger DMSO concentrations. This observation suggests that up to 30% DMSO only limited conformational modifications take place, ascribable to the aforementioned decrease of the dielectric constant and related changes in the shielding of electrostatic interaction and in the reduction-induced solvent reorganization. On the contrary, at larger DMSO concentrations a more significant structural change must occur beyond solvent effects.

**Figure 5:**
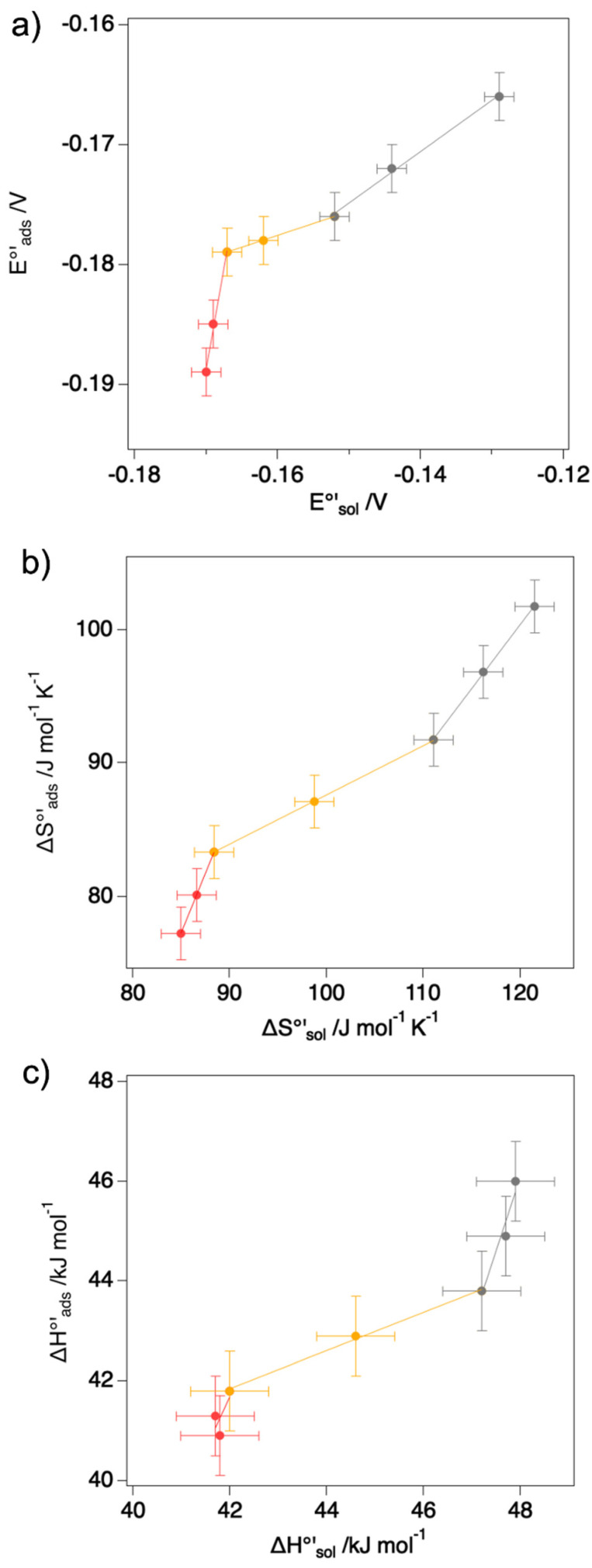
Comparison of thermodynamic parameters (E°′, ΔH°′_rc_ and ΔS°′_rc_ in panels (**a**–**c**), respectively) obtained for the adsorbed M80A cytc variant and for the same species in solution at increasing DMSO concentration. Red points refer to 0% and 5% DMSO; orange points refer to 10% and 20% DMSO; gray points to 30%, 40% and 50% DMSO. The data corresponding to the SAM-immobilized proteins are taken from ref. [44]. The red, orange and gray lines are least-squares fits to the data points for %DMSO ≤ 10%, 10% ≤ %DMSO ≤ 30% and DMSO ≥ 30%.

A biphasic H-S compensation plot was also reported previously for the MUA/MU immobilized M80A [44], but the change in slope occurred at a different DMSO concentration and a poorer H-S compensation was observed. This indicates that the effect of the organic solvent on the reduction thermodynamics is indeed modulated by the conditions under which the redox process takes place, namely whether the protein is in the adsorbed or freely diffusing state. To further investigate this aspect the E°′, ΔH°′_rc,_ and ΔS°′_rc_ values for the reduction process measured at different mixed solvent compositions for the adsorbed M80A were plotted against the corresponding values for the freely diffusing species (Figure 5). Invariably, three well-defined regions can be identified. The first region spans the 0–5% DMSO range and is characterized by a slope > 1 for all the quantities: this indicates that the DMSO-induced change is more relevant for the adsorbed protein than in the solution state. For %DMSO between 10 and 30 the trend is opposite, with a slope that is significantly < 1 for all the quantities, suggesting that, in this concentration range, the organic solvent-induced effects are more relevant for the protein in solution than for the adsorbed species. For %DMSO > 30%, E°′ still shows a slope < 1, but larger with respect to that of the 10–30% region (0.43 vs. 0.20): this effect is mostly contributed by the ΔH°′_rc_ change, which is definitely larger for the immobilized protein, while the ΔS°′_rc_ variation induced by DMSO are almost the same for the two conditions.

Some hypotheses can be put forward to try to elucidate the molecular determinants of the different responses of solution and adsorbed M80A to the presence of increasing concentrations of DMSO. As mentioned above, the main effect of the organic solvent is the modification of the apparent dielectric constant, which alters the electrostatic effects due to the protein charges. When the protein is immobilized on an anionic SAM, additional interactions between the M80A surface positive charges and the negatively charged carboxylate groups of the SAM must be taken into account [28,35,36,39]. From the larger effect exerted by the medium on M80A in solution vs. the immobilized species for %DMSO > 10, it appears that the latter interaction is affected to a lesser extent by the water/DMSO ratio than the electrostatic effects due to the protein charges in freely diffusing conditions. The discontinuity observed around 30% DMSO might be due, at least in part, to a structural transition, probably involving the substitution of axial OH^−^ ligand by a second histidine [30,37,38,39,49]. As for the slopes observed at the lowest DMSO concentrations, one possibility is that the water solvates DMSO making it less effective in modulating the apparent dielectric constant, while in the same concentration range the interaction between the organic molecules and the SAM headgroups is more relevant in modulating the thermodynamic parameters.

### 2.2. Effect of DMSO on the ET Kinetics

The rate constants k_ET_ for the ET process between freely diffusing M80A and the electrode were determined with the procedure of Gulaboski et al. [66] (Table 2) and compared with the corresponding values for the immobilized protein determined previously [44]. k_ET_ is the heterogeneous electron transfer rate constant, calculated at the standard reduction potential, for second-order reactions in which the ET species is freely diffusing in solution. The activation enthalpy values ΔH^#^ and the pre-exponential term A were obtained according to the Arrhenius equation k_ET_ = A exp(−ΔH^#^/RT), namely from the slope of the ln(k_ET_) vs. 1/T plot obtained from the temperature dependence of the rate constant k_s_ in the 5–35 °C range using a non-isothermal cell [38,39,49,67,68] (Figure S2 and Table 2).

The k_ET_ values decrease from 0 to 30% DMSO but remain constant at higher DMSO concentrations. Although the k_ET_ values cannot be directly compared to the corresponding values of ET rate constants for the immobilized protein k_s_ (see Table 2), since k_ET_ and k_s_ refer to two kinetic processes of a different order, it is apparent that both constants decrease as the DMSO concentration increases, although such decrease is percentually more relevant for the protein in solution than for its adsorbed counterpart. The ET rate in cytc is known to be affected by the first coordination sphere of the metal center, the protein conformation in the vicinity of the heme, and the solvent accessibility to the heme pocket [20]. Analysis of the DMSO dependence of ΔH^#^ and lnA would help gain insight into the determinants of the ET rate changes. In aqueous solutions and for folded cytc, the activation entropy ΔS^#^ is often considered negligible [14,30,49,64,67,69,70,71]: this assumption, though, is hardly acceptable when dealing with organic solvents that likely cause conformational changes leading to partial unfolding and increased access of water and DMSO molecules to the heme center. In particular, the presence of solvent molecules in the catalytic pocket is known to be a major determinant of ΔS^#^ [49,70,72]. As the heterogeneous ET constant k_ET_ can be expressed as:

(1) $$k_{\mathrm{ET}}=A^{\prime }\cdot e^{\left({\Delta S}^{\#}/R\right)}\cdot e^{\left(-{\Delta H}^{\#}/\mathrm{RT}\right)}$$

when discussing the effect of DMSO on the ET kinetics, we therefore will focus on the pre-exponential factor A = A′exp(ΔS^#^/R) as a whole.

**Figure 6:**
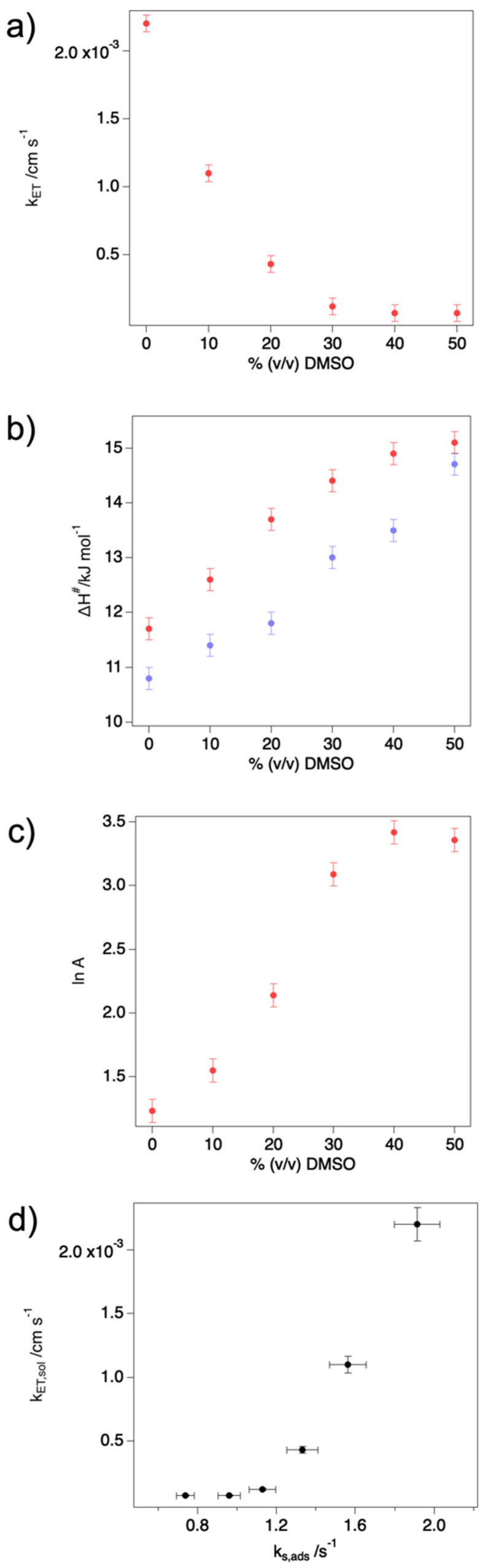
Dependence of k_ET_ (**a**), ΔH^#^ (**b**) and lnA (**c**) vs. % (*v*/*v*) DMSO. In panel (**b**), the red dots refer to the protein in solution, while the lilac ones to the protein immobilized on a SAM. (**d**) Plot of the ET rate constant in solution (k_ET_) vs. the corresponding value, at the same DMSO concentration, for the protein immobilized on a SAM (k_s_). The data corresponding to the SAM-immobilized proteins in panels (**b**,**d**) are taken from ref. [44].

From the data in Table 2 and the plots in Figure 6, it is apparent that the contributions to k_ET_, namely ΔH^#^ and lnA, increase progressively with increasing DMSO concentration, following a bimodal trend: these two quantities have the opposite effect on the k_ET_ value, with ΔH^#^ prevailing over the pre-exponential factor, leading to a decrease of the rate constant with increasing organic solvent content. In particular, both ΔH^#^ and lnA vary significantly for DMSO concentration < 40%, while they are basically unaltered at higher %DMSO as observed for k_ET_.

This behavior is qualitatively similar to that observed for M80A in mixed H_2_O/DMSO solvent immobilized on an anionic SAM. Nevertheless, some significant differences do exist. Indeed, for DMSO content below 50%, the ΔH^#^ values are larger for the protein in solution suggesting that the ET reorganization energy might change with markedly different dependence on DMSO concentration for the freely diffusing and adsorbed protein. Moreover, the plot of the ET rate constant values obtained for the adsorbed M80A (k_s_) vs. those calculated here for the same species in solution (k_ET_), at increasing DMSO concentration (Figure 6d), also displays a bimodal trend. In the low-DMSO region (from 0 to 20%) roughly linear relationship between k_s_ and k_ET_ is observed, indicating that the effect of the solvent is proportional to DMSO concentration for both rate constants. On the contrary, in the high-DMSO region (from 30 to 50%) k_ET_ is basically unaffected by DMSO concentration while k_s_ keeps decreasing. This indicates that for k_s_ at high DMSO concentration the solvent likely plays a role in the interaction between the protein and the SAM on which it is adsorbed, rather than causing structural changes on the protein, as these, if present, would also impact on k_ET_.

### 2.3. Effect of DMSO on the Structural Properties of M80A

We combined electronic absorption, (magnetic) circular dichroism, and fluorescence emission spectra to investigate the effects of increasing DMSO concentration on M80A, in the same range explored electrochemically. In particular, we focused on: (i) the overall three-dimensional structure of the protein, (ii) the structure of the heme environment, and (iii) the electronic properties of the heme group.

**Figure 7:**
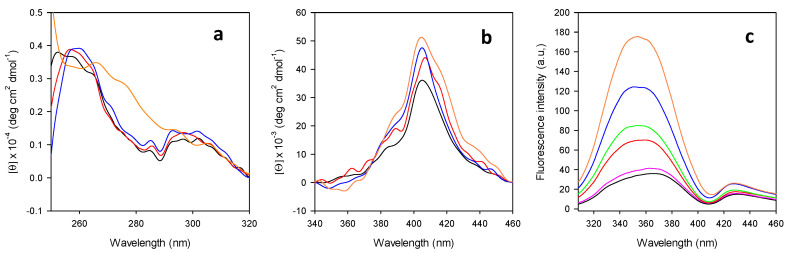
Spectra of M80A cytc at increasing DMSO concentration in 10 mM phosphate buffer, pH 7.0. (**a**) Near-UV CD; (**b**) CD in the visible region; (**c**) Fluorescence emission. Black: 0% DMSO; pink: 10% DMSO; red: 20% DMSO; green: 30% DMSO; blue: 40% DMSO; orange: 50% DMSO.

Insight into the effects of DMSO on the overall structure of the M80A cytc mutant was gained from the CD spectra in the near UV region (250–350 nm) and by fluorescence emission by Trp59 (Figure 7a,c, respectively). The near UV CD spectrum features two narrow minima at 282.5 and 289 nm (ascribable to transitions that involve the Trp59 lateral chain) [37,73,74,75,76] and three maxima between 250 and 270 nm (arising from transitions involving the Tyr residues side chains and the heme group [37,73,75]. Such spectrum is basically superimposable to that of wt cytc in the absence of DMSO, confirming that the three-dimensional structure of the protein is not significantly affected by the substitution of the Fe-coordinating methionine with a non-coordinating alanine [37,74,75,77]. For DMSO up to 40%, the 282.5 and 289 nm bands undergo minor changes in intensity and position, while they disappear at 50%, suggesting that only the highest DMSO concentration studied significantly alters the position of Trp59 with respect to the heme group.

This finding is confirmed by the analysis of the fluorescence emission spectra of M80A (Figure 7c), which are commonly used to investigate structural rearrangements of cytochrome *c* [74,76,78]. In the folded protein, the emission of the single Trp59 is quenched by the heme. Hence, as Trp59 moves away from the heme due to protein unfolding, a corresponding increase in fluorescence emission is observed [74,76,78].

The fluorescence emission of Trp59 in M80A in a fully aqueous solvent is quenched as observed in the wt protein, thereby confirming that deletion of the axial methionine does not influence the overall 3D structure of the protein. Nevertheless, the maximum of the emission is significantly red-shifted (367 nm) compared to the wt species (330 nm) in line with the increased solvent exposure of Trp59 upon mutation [74,76,79]. Upon gradual increase in DMSO concentration, an increase in intensity and a blue shift of the emission maximum down to 354 nm occurs, indicating an increased distance between Trp59 and the heme group and that the surroundings of Trp59 become progressively less polar (Table 3). These data suggest that DMSO concentrations as high as 50% are needed to start a modification of the three-dimensional structure of the M80A mutant. The changes observed at lower DMSO concentrations might be ascribed to a progressive DMSO-induced decrease in the dielectric constant of the medium that might lead to increased electrostatic repulsions between the positively charged, solvent-exposed aminoacidic side chains, resulting in a gradual increase of the molecular volume, as was hypothesized for bovine cytc under the same experimental conditions [56].

We then restricted our attention to the protein cavity surrounding the heme group, which can be investigated by following the DMSO-induced changes in the CD spectra in the Soret region (Figure 7b). In fact, the optical activity in this spectral region depends on the coupling between electric dipole transition moments of π→π* transitions of the heme group and of the aromatic ring of Phe82 and is therefore affected by the interactions between the heme itself and its surroundings [27,76,80,81,82,83,84]. The CD spectrum of the M80A mutant in phosphate buffer differs from that of wt cytc in both the red shift of the maximum (406 vs. 400 nm) and in the absence of the minimum located between 415 and 420 nm which provides evidence for the substitution of axial ligand Met80 by the OH^−^ ion [27,76,80,81,85]. The overall shape of the CD spectrum in the Soret region is not significantly altered by the presence of the organic solvent, although its intensity increases between 0 ≤ %DMSO ≤ 10 and above 30%DMSO. However, no systematic, monotonic change with increasing DMSO concentration is also observed in the corresponding difference CD spectra.

**Figure 8:**
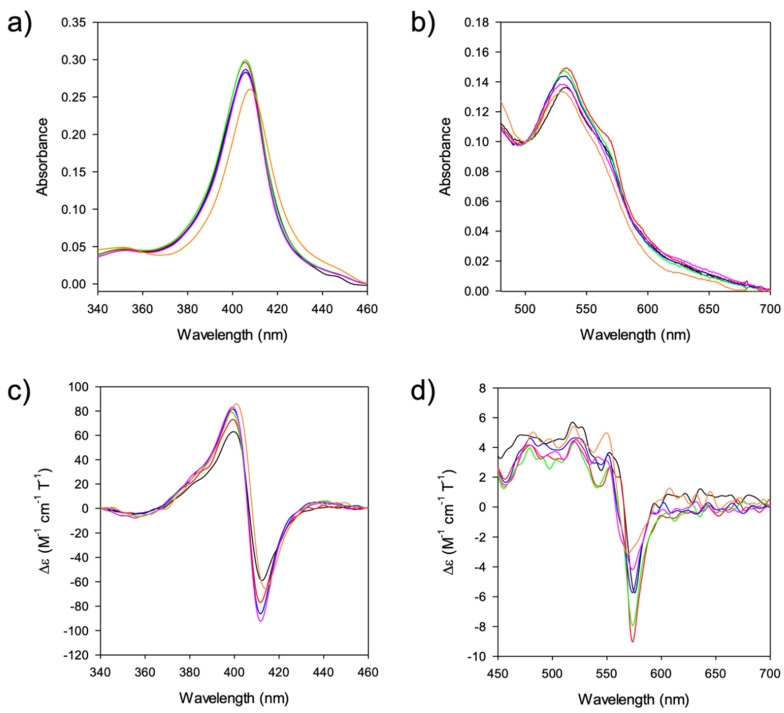
Spectra of M80A cytc at increasing DMSO concentration in 10 mM phosphate buffer, pH 7.0. (**a**) Electronic absorption in the Soret region; (**b**) Electronic absorption in the visible region; (**c**) MCD in the Soret region; (**d**) MCD in the visible region. Black: 0% DMSO; Red: 10% DMSO; Green: 20% DMSO; Blue: 30% DMSO; Pink: 40% DMSO; Orange: 50% DMSO.

The effects of DMSO on the electronic properties of the heme group were then investigated by both electronic absorption spectroscopy and MCD. In the absence of DMSO, the electronic absorption spectrum of M80A (Figure 8a) features a symmetric Soret band at 406 nm, while the corresponding MCD S-shaped signal (Figure 8c) displays a maximum at 399 nm, a minimum at 412 nm, and a crossover point at 406 nm [37]. The position of both signals is unaltered by the presence of %DMSO ≤ 40, shifting to slightly higher wavelengths at %DMSO = 50. In the visible region (470–700 nm), the electronic absorption spectrum recorded in an aqueous buffer features a maximum at 533 nm and a shoulder at 570 nm, while an S-shaped signal (with minimum, maximum, and crossover points at 575, 552 and 566 nm, respectively) is observed in the MCD spectrum [37] (Figure 8d). Their position is almost unaffected up to 40% DMSO, while a blue shift is observed in the presence of 50% DMSO (Figure 8b,d, Table 3). Moreover, a significant change in the shape of the MCD signal occurs in the presence of 50% DMSO (Figure 8d, Table 3).

The electronic absorption and MCD spectra of ferric M80A at pH 7 in the absence of DMSO (Figure 8 and Table 3) match those reported previously [34,37], indicating a 6-coordinate His/OH^−^ low spin heme [31,32,33,34,35,36,38]. The limited spectroscopic changes observed up to 40% DMSO indicate that no significant change in the heme electronic properties and axial coordination occurs and probably arise from reorganization effects in the surroundings of the heme, induced by the decrease of the solution dielectric constant.

The significant spectroscopic changes observed at 50% DMSO suggest the onset of a transition towards another low spin form, most likely as a consequence of the replacement of the axial OH^−^ by a different ligand, as observed upon urea denaturation [37].

## 3. Materials and Methods

**Materials.** All chemicals were reagent grade. 4-mercapto-pyridine was purchased from Sigma and used without further purification. Water was purified through a Milli-Q Plus Ultrapure Water System coupled with an Elix-5 Kit (Millipore). The water resistivity was over 18 MΩ cm. Anhydrous DMSO (dried, water content ≤ 0.02%) was purchased by Sigma-Aldrich and used without further treatment. Solvent compositions are expressed in percent by volume.

**Protein Production and Isolation**. The nontrimethylated M80A mutant of recombinant *S. cerevisiae* iso-1 cytochrome *c* was expressed and isolated as described previously [29,34,35,36]. To prevent protein dimerization, the variant features the C102T mutation [29,34,35,36].

**Spectroscopic Measurements.** Electronic absorption, CD and MCD spectra were recorded with a Jasco J-810 spectropolarimeter. The magnetic field was provided by a GMW Magnet system Model 3470 split coil superconductivity magnet with a maximum field of 1 Tesla (T). Both CD and MCD spectra were measured in *θ* = mdeg.

CD and MCD spectra were converted, respectively, to molar ellipticity [*θ*] and ∆*ε* [M^−1^ cm^−1^ T^−1^] using the following conversion factors

(2) [θ]=θ(deg)·100/(d·c)

(3) Δϵ=θ/(32980·c·d·B)

where *c* is the protein concentration (mol/dm^3^), *d* is the thickness of the sample (path length, 0.5 cm) and *B* is the magnetic field (1 T) [25,34,37,74,75,77,86].

All experiments were carried out at 25 °C with mixed water/DMSO protein solutions freshly prepared before use, by adding to a known volume of a concentrated protein solution in 10 mM phosphate buffer pH 7 the required volume of anhydrous DMSO. The final volumes of all solutions were made the same by adding known volumes 10 mM phosphate buffer pH 7. Protein concentration was checked spectrophotometrically, using ε_406_ = 121,700 M^−1^ cm^−1^ [31,32].

**Electrochemical Measurements.** Square wave voltammetries (SWV) were carried out with a Potentiostat/Galvanostat mod. 273A (EG&G PAR, Oak Ridge, TN, USA), using a cell for small volume samples (0.5 mL) under argon.

A polycrystalline gold wire functionalized with 4-mercapto-pyridine [37,87], a platinum sheet, and a saturated calomel electrode (SCE) were used as the working, counter, and reference electrodes, respectively. The electric contact between the SCE and the working solution was achieved with a Vycor^®^ (from PAR) set. Reduction potentials were calibrated against ferrocene/ferrocenium couple under all experimental conditions employed in this work to make sure that the effects of liquid junction potentials were negligible.

Mixed water/DMSO protein solutions were freshly prepared before use, by adding the required volume of anhydrous DMSO to a known volume of a concentrated protein solution in 5 mM phosphate buffer plus 100 mM sodium perchlorate at pH 7. The final volumes of all solutions were made the same by adding known volumes of 5 mM phosphate buffer containing 100 mM sodium perchlorate at pH 7. Protein concentration was checked spectrophotometrically, using ε_406_ = 121,700 M^−1^ cm^−1^ [31,32].

The formal potentials E°′ were calculated as the semisum of the anodic and cathodic peak potentials obtained by SWV performed in anodic and cathodic scan, respectively (Figure 2). Signals persist for several SWV throughout the temperature range investigated. The experiments were performed at least five times and the E°′ values were found reproducible within ±0.002 V. Effects of uncompensated cell resistance were minimized using the positive feedback iR compensation function of the potentiostat, set at a value slightly below that at which current oscillations emerge [44,88].

Variable-temperature SWV experiments (Figure S3) were carried out using a “non-isothermal” cell, in which the reference electrode was kept at constant temperature (21 ± 0.1 °C) whereas the half-cell containing the working electrode and the Vycor^®^ junction to the reference electrode was under thermostatic control with a water bath [59,89,90,91,92]. The temperature varied from 5 to 35 °C. With this experimental configuration, the standard entropy change for heme Fe(III) to Fe(II) reduction in cytc (∆S°′_rc_) is given by [59,89,90,91,92]:

(4) $$\Delta {{S{}^{\circ}}^{\prime }}_{\mathrm{rc}}={{S{}^{\circ}}^{\prime }}_{\mathrm{red}}-{{S{}^{\circ}}^{\prime }}_{\mathrm{ox}}=\mathrm{nF}\left(\frac{{\mathrm{dE}{}^{\circ}}^{\prime }}{\mathrm{dT}}\right)$$

thus, ∆S°′_rc_ was determined from the slope of the plot of E°′ versus temperature which turns out to be linear under the assumption that ∆S°′_rc_ is constant over the limited temperature range investigated. With the same assumption, the enthalpy change (∆H°′_rc_) was obtained from the Gibbs-Helmholtz equation, namely as the negative slope of the E°′/T versus 1/T plot [8,59,92]. The nonisothermal behavior of the cell was carefully checked by determining the ∆H°′_rc_ and ∆S°′_rc_ values of the ferricyanide/ferrocyanide couple.

The rate constants k_ET_ were calculated using the procedure developed by Gulaboski et al. to investigate the redox kinetics of freely diffusing proteins using the half-peak width (ΔE_p/2_) of the corresponding square-wave voltammograms. [66]. The k_ET_ values were averaged over five measurements and found to be reproducible within 6%, which was taken as the associate error.

The k_ET_ values were measured in the range of 5–35 °C to determine the activation enthalpies (ΔH^#^) using the Arrhenius equation.

## 4. Conclusions

We analyzed the effect of DMSO on both functional (electrochemical) and structural properties of the M80A cytc mutant. In general, the molecular properties are not dramatically altered by the organic solvent in the investigated concentration range, and the main effects are probably to be ascribed to changes in the solvent properties rather than to dramatic structural modifications that would also result in severely altered thermodynamics and kinetics of ET.

Our analysis of thermodynamics and kinetics of reduction suggests that the three-dimensional structure and the first coordination sphere of the metal center undergo limited changes in the explored DMSO concentration range, characterized by a progressive modification of the ET features without the appearance of novel signals that might suggest the appearance of a novel species. These changes are most likely to be ascribed mainly to a modified value of the dielectric constant as the DMSO concentration increases. Overall, the M80A variant in solution seems to be more markedly affected by the increase of the organic fraction in the mixed solvent than its adsorbed counterpart, especially with respect to the activation enthalpy contribution to the ET kinetics. Concerning the ET thermodynamics, immobilized M80A seems to be more sensitive to medium effect than the diffusing form at DMSO concentration < 10%, while the opposite occurs at larger DMSO content.

This overall picture is supported by spectroscopic investigations: in general, at % DMSO between 0 and 40, the spectral features remain the same, and only minor intensity changes are observed. These findings indicate that neither the coordination set nor the electronic properties of the metal center undergo major changes. Therefore, despite the potential capability of DMSO to bind the heme iron, the latter remains hexacoordinated and low-spin, with the axial positions occupied by the proximal histidine and an OH^−^ ion. The small spectral changes observed are most likely the result of reorganization effects in the vicinity of the heme, caused by the decrease of the solution dielectric constant. The overall structure of the protein is also maintained, although a variation of the molecular volume might be inferred from the CD spectra in the near UV and the emission fluorescence changes induced by DMSO. On the contrary, 50% DMSO seems to cause more significant, though not dramatic, spectroscopic changes: possibly, at this concentration, the onset of a transition towards another species might occur. The latter, despite remaining low-spin, most likely features substitution of the axial OH^−^ by a different ligand.

Our findings confirm the possibility of employing the M80A mutant in (nano)biotechnological applications such as biosensing and biocatalysis also in mixed water/DMSO solvents, therefore expanding the number of potential substrates to be transformed or detected by exploiting the M80A peculiar (electro)catalytic properties that are absent in wt cytc.

## Figures and Tables

**Table 1:** Reduction thermodynamics for the M80A cytc variant in mixed water/DMSO solutions at increasing DMSO content [^a^].

% DMSO (*v*/*v*)	E°′ (V)	ΔS°′_rc_ (J mol^−1^ K^−1^)	ΔH°′_rc_ (kJ mol^−1^)
0	−0.170	85.0	41.8
5	−0.169	86.6	41.7
10	−0.167	88.4	42.0
20	−0.162	98.8	44.6
30	−0.152	111.1	47.2
40	−0.144	116.2	47.7
50	−0.129	121.5	47.9

^a^ E°′ obtained at 20 °C. The average errors on E°′, ΔS°′_rc_ and ΔH°′_rc_ are ±0.002 V, ±2 J K^−1^mol^−1^, ±0.8 kJ mol^−1^, respectively. Working solution: 5 mM sodium phosphate buffer pH 7 plus 100 mM sodium perchlorate.

**Table 2:** Redox kinetics of the M80A mutant of yeast cytc in the presence of increasing DMSO concentration. k_ET_ was measured at 20 °C [^a^].

% DMSO (*v*/*v*)	k_ET_ (cm s^−1^)	ΔH^#^ (kJ mol^−1^)	lnA	k_s_^b^ (s^−1^)	ΔH^# b^ (kJ mol^−1^)	lnA ^b^
0	0.00220	11.7	1.23	1.91	10.8	5.09
10	0.00110	12.6	1.55	1.56	11.4	5.11
20	0.00043	13.7	2.14	1.33	11.8	5.13
30	0.00012	14.4	3.09	1.13	13.0	5.45
40	0.00007	14.9	3.42	0.96	13.5	5.50
50	0.00007	15.1	3.36	0.74	14.7	5.72

^a^ The average errors on k_ET_, ΔH^#^ and lnA are ±6%, ±0.2 kJ mol^−1^ and 0.09, respectively. Working solution: 5 mM phosphate buffer pH 7 plus 100 mM sodium perchlorate. ^b^ for the M80A variant immobilized on an anionic mercaptoundecanoic acid/mercaptoundecanol SAM, from ref. [44].

**Table 3:** Wavelengths (nm) of the relevant spectral features of the UV-vis, MCD, CD and fluorescence emission spectra for the M80A variant of *Saccharomyces cerevisiae* iso-1 cytochrome *c* in 10 mM phosphate buffer pH 7, in the presence of increasing concentrations of DMSO.

% DMSO (*v*/*v*)	Absorption		MCD						CD	Fluorescence
	Soret	Vis	Soret			Vis			Soret	
			Peak	Zerocross	Trough	Peak	Zerocross	Trough	Peak	
0	406	533, 570(sh) ^a^	400	406	412	552	566	575	405	364
10	406	533, 570(sh) ^a^	399	406	412	553	566	574	406	363
20	406	533, 570(sh) ^a^	399	406	412	553	560	574	407	359
30	406	532, 570(sh) ^a^	399	406	412	551	559	573	406	356
40	406	532, 570(sh) ^a^	399	406	412	551	558	573	405	351
50	408	530, 561(sh) ^a^	401	408	414	550	561	569	405	354

^a^ sh stands for shoulder.

## Data Availability

The date presented in this study are available on request from the corresponding authors.

## Supplementary Materials

The following supporting information can be downloaded at: https://www.mdpi.com/article/10.3390/molecules27175630/s1, Figure S1: E°′ vs. T plot of M80A cytc at different DMSO percentage; Figure S2: Arrhenius plots of M80A cytc at different DMSO percentage; Figure S3: Square wave voltammograms of M80A cytc in 50% DMSO at 278K and 303K.
