# Supplementary material for: Assessing the Functional and Structural Stability of the Met80Ala Mutant of Cytochrome c in Dimethylsulfoxide
Source: Molecules. 2022 Aug 31;27(17):5630. doi: 10.3390/molecules27175630 (PMC9458088; doi:10.3390/molecules27175630)
Supplement: Supplementary file 1 [file molecules-27-05630-s001.zip › molecules-1817181-supplementary.pdf]

Supporting Information

Assessing the functional and structural stability of the Met80Ala mutant of cytochrome *c* in dimethylsulfoxide

Giulia Di Rocco [1], Antonio Ranieri [1], Marco Borsari [2], Marco Sola [1], Carlo Augusto Bortolotti [1,*], Gianantonio Battstuzzi [2,*]

[1] Department of Life Sciences, University of Modena and Reggio Emilia, via Campi 103, 41125 Modena, Italy
[2] Department of Chemistry and Geology University of Modena and Reggio Emilia, via Campi 103, 41125 Modena, Italy
Correspondence: carloaugusto.bortolotti@unimore.it; Tel. 00390592058608 (CAB), gianantonio.battistuzzi@unimore.it; Tel. 0039059208639 (GB)

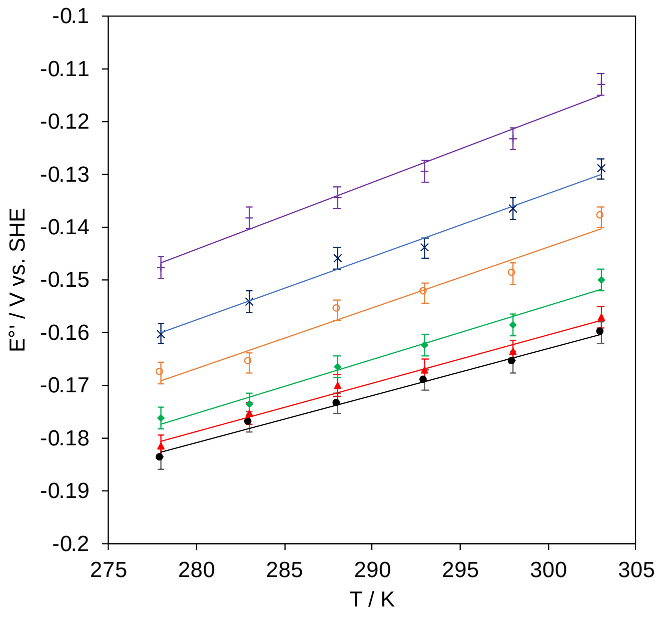

Figure S1. E°′ vs. T plots of M80A cytc variant in 5% (black) 10% (red) 20% (green) 30% (orange) 40% (deep blue) and, 50% (violet) percentage (v/v) of DMSO. Base electrolyte: 5 mM sodium phosphate buffer pH 7 plus 100 mM sodium perchlorate.

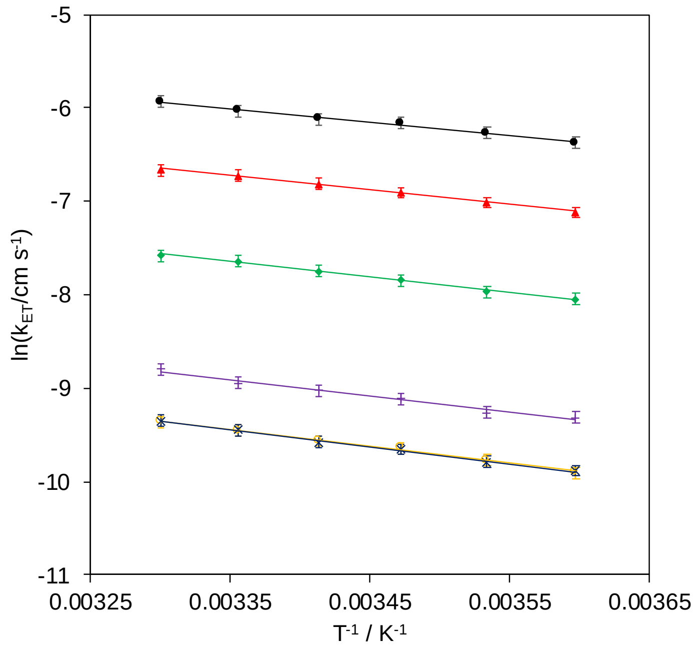

Figure S2. Arrhenius plots of M80A cytc variant in 0% (black) 10% (red) 20% (green) 30% (violet) 40% (yellow) and, 50% (deep blue) percentage (v/v) of DMSO. Base electrolyte: 5 mM sodium phosphate buffer pH 7 plus 100 mM sodium perchlorate.

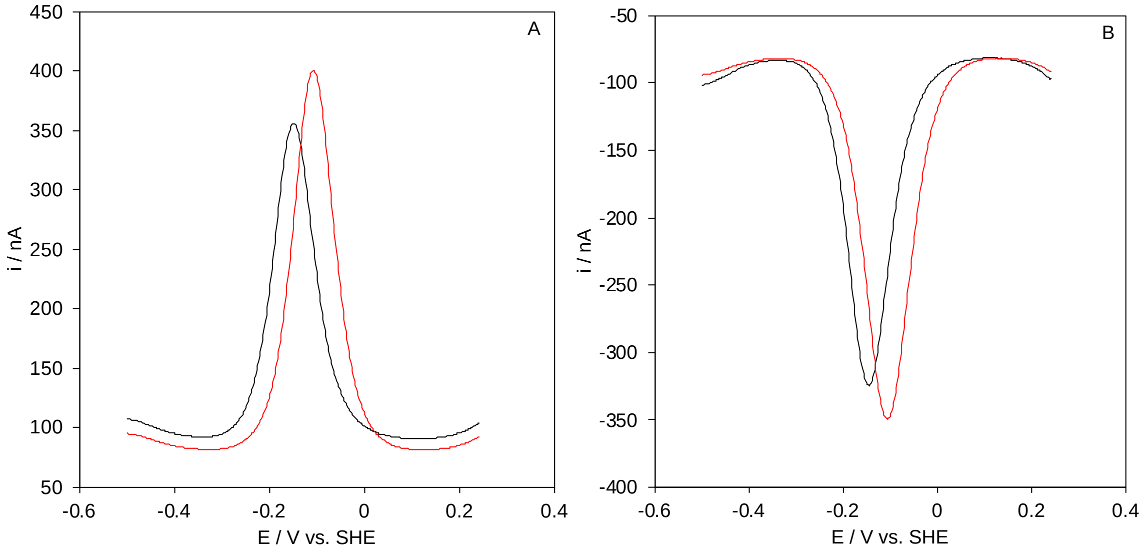

Figure S3. Cathodic (A) and anodic (B) Square Wave Voltammograms of the M80A cytc variant in 50% DMSO at 278 K (black line) and 303 K (red line). Base electrolyte: 5 mM sodium phosphate buffer pH 7 plus 100 mM sodium perchlorate.
